# Clear cell variant of follicular thyroid carcinoma with normal thyroid-stimulating hormone value: a case report

**DOI:** 10.1186/1752-1947-8-160

**Published:** 2014-05-22

**Authors:** Ilyas Sayar, Kemal Peker, Ibrahim Gelincik, Levent Demirtas, Arda Isik

**Affiliations:** 1 Department of Pathology Faculty of Medicine, Erzincan University, 24100 Erzincan, Turkey; 2 Department of General Surgery Faculty of Medicine, Erzincan University, 24100 Erzincan, Turkey; 3 Department of Pathology Faculty of Medicine, Namik Kemal University, 59100 Tekirdag, Turkey; 4 Department of Internal Medicine Faculty of Medicine, Erzincan University, 24100 Erzincan, Turkey

## Abstract

**Introduction:**

Clear cell carcinomas of the thyroid gland with normal thyroid-stimulating hormone value are very rare, but clear cell changes are described in most reported cases of thyroidal lesions.

**Case presentation:**

In this report, we describe the case of a 50-year-old Caucasian woman with a normal thyroid-stimulating hormone level who underwent surgery to treat a multi-nodular goiter. The pathology was a clear cell variant of follicular thyroid carcinoma. The tumor was 1cm in diameter and consisted of pure clear cells.

**Conclusion:**

Clear cell variants of follicular thyroid carcinoma are rarely seen, especially it is misdiagnosed with metastatic renal cell carcinoma. In this report, we describe the case of a patient with a clear cell variant of follicular thyroid carcinoma with an interesting pathology.

## Introduction

Thyroid follicular carcinoma is a malignant epithelial tumor comprising approximately 10% to 15% of thyroid carcinomas. It occurs more commonly in women, and the World Health Organization divides it into two groups based on histology: the oncocytic variant and the clear cell variant [1]. Clear cell carcinomas of the thyroid gland are very rare, but clear cell changes have been described in most reported cases of thyroidal lesions. In our present case report, we describe the case of a patient with the clear cell variant of follicular thyroid carcinoma, which is rarely seen, and primarily existed as the view of metastatic renal cell carcinoma (RCC). This case is important because the tumor was 1cm in diameter and consisted of pure clear cells together with a multi-nodular goiter.

## Case presentation

A 50-year-old Caucasian woman was admitted to our hospital with complaints of swelling and pain in the neck. A multi-nodular goiter was discovered during a routine examination of the patient. She had a history of fullness of the neck for about 3 months. During palpation, a right thyroid lobe growth of slightly stiff consistency and moving nodules about 1.5cm in diameter were detected. Her systemic examination was normal. No anomalies were found her biochemical test results. Multiple hypoechoic bilateral nodules, the largest of which was on the right side (13 × 10mm diameter), were seen on the neck ultrasound. Insufficient tissue material was obtained by fine-needle aspiration biopsy due to the hard consistency of the right lobe nodule. Total thyroidectomy was performed on the basis of a pre-surgical diagnosis of multi-nodular goiter.

**Figure 1:**
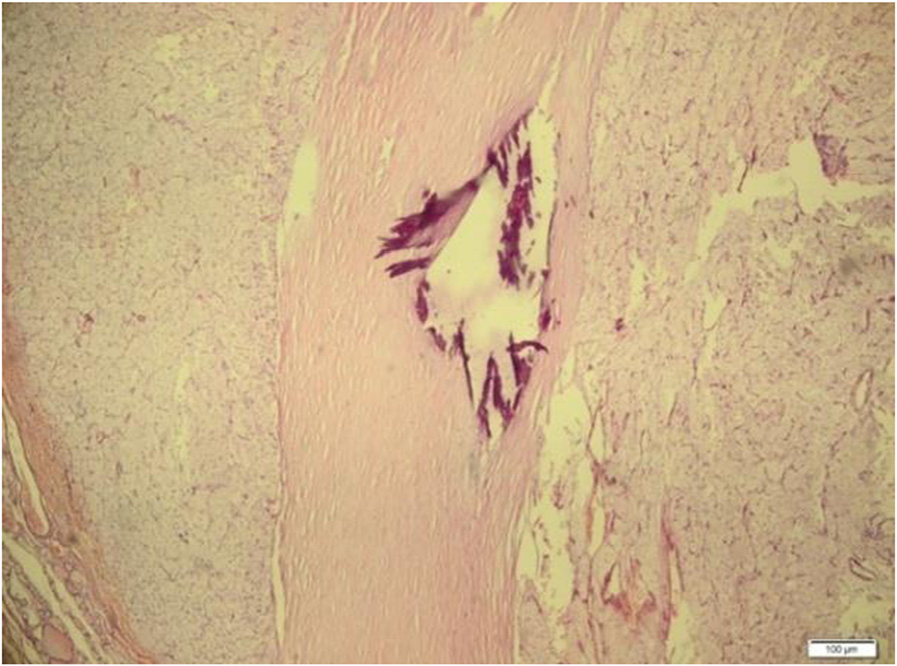
**Histological tissue specimen separated by a thick capsule.** (Hematoxylin and eosin stain; original magnification 40×).

**Figure 2:**
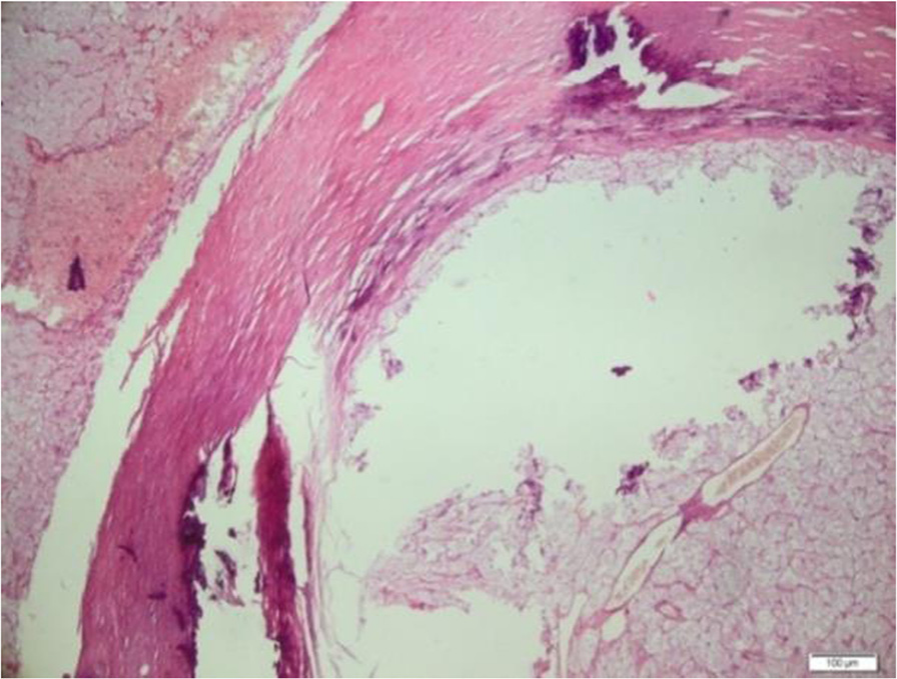
**Histological tissue specimen of the left side showing extracapsular extension separated by a thick capsule.** Pushed capsule was monitored. (Hematoxylin and eosin stain; original magnification, 40×).

**Figure 3:**
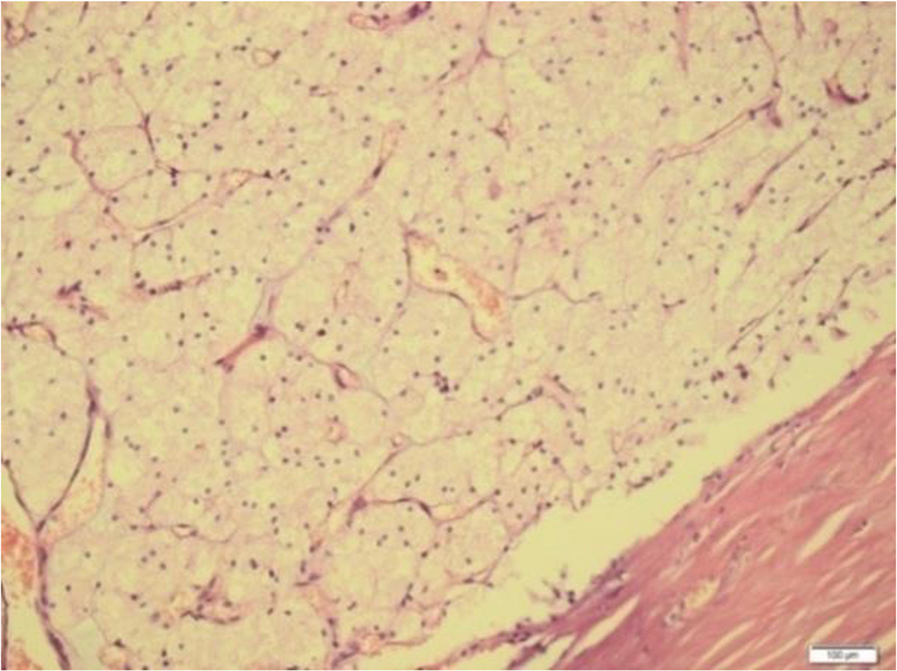
**Histological tissue specimen showing thyrocytes, in which the nucleus settled at the center, with a thick capsule at the bottom and clear cytoplasm.** (Hematoxylin and eosin stain; original magnification, 200×).

**Figure 4:**
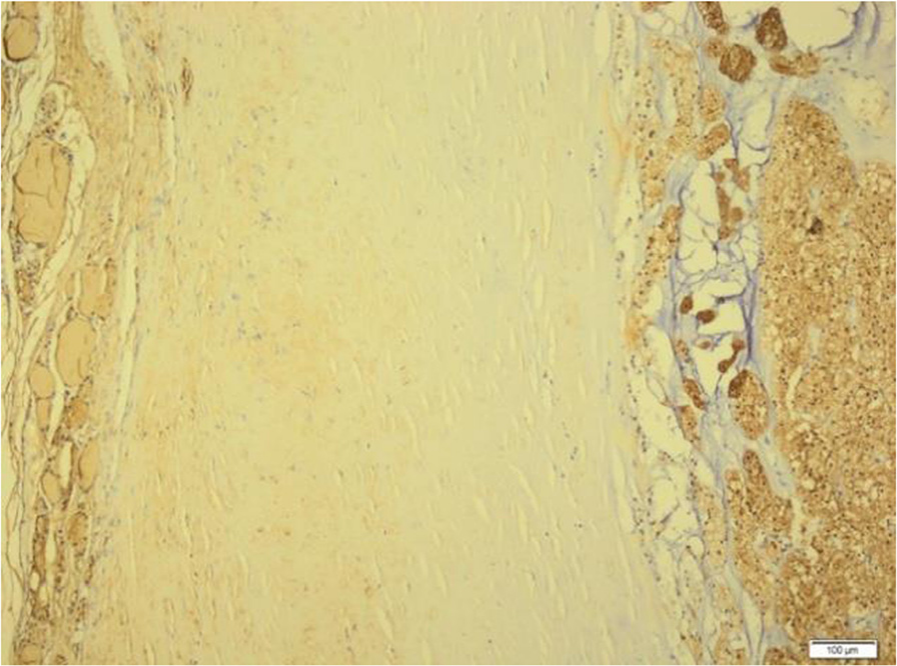
**Histological tissue specimen showing thick capsule in the middle, tumor formation at right and normal thyroid tissue at left.** (Thyroglobulin stain; original magnification, 100×).

During macroscopic examination, we observed burgundy red elastic thyroidectomy material. This mass weighed 25g, and its size was 4cm × 3cm × 2cm in the right lobe and 5cm × 4cm × 2 cm in the left lobe. In the right lobe, which appeared moderately rich in colloid on the cross-sectional image, we observed a thickly encapsulated area 1cm in diameter that stained as a solid beige color with hematoxylin and eosin. We also observed nodular structures 0.5cm and 0.3cm in diameter, in the left lobe.In the light microscopy, thick capsulated tumoral areas which had cells with clear cytoplasm and nucleus usually located at the center (Figure 1), pushed the capsule in some points of view, and forms outside the capsule in two focuses were observed (Figure 2). Colloid in tumor tissue was too little (Figure 3). There was no lymphovascular invasion. Thyroid tissue except tumor was in accordance with nodular hyperplasia. In the differential diagnosis, tumor cells stained positive for thyroglobulin with immunohistochemical staining especially considering the areas of clear cell tumor (Figure 4). Vimentin, RCC, synaptophysin, and chromogranin staining were not observed. The case was reported as clear cell variant of follicular thyroid carcinoma.

## Discussion

The appearance of thyroid follicular carcinomas is round or ovoid, generally encapsulated and larger than 1cm in diameter. Clinically, follicular carcinomas are usually asymptomatic. Symptoms such as dyspnea and dysphagia are rarely observed. They are most often seen in large, invasive follicular carcinomas. On scintigraphic imaging scans, they are seen as cold nodules similar to papillary carcinoma nodules. Histopathologically, capsular and vascular invasion are diagnostic. Metastasis in oncocytic follicular carcinomas has been reported to be more common than in the non-oncocytic type [1-3].

Cytoplasmic clear cell changes can occur in papillary and follicular carcinomas, but the clear cells are not usually pure. Clear cell variants of follicular thyroid carcinomas consist of clear cytoplasmic cells that contain significantly enlarged mitochondria and endoplasmic reticulum. Other authors have previously reported that the cellular changes that occur in clear cytoplasm in thyroid neoplasia were caused by glycogenation, intracellular accumulation of thyroglobulin, dilatation and hypertrophy in mitochondrial cells, and Golgi complex hypertrophy due to excessive secretion of thyroid-stimulating hormone (TSH) [1,2]. In our patient, the pre-operative TSH and free T3 and T4 thyroid hormone levels were normal.

The differential diagnosis of the clear cell variant of follicular carcinoma includes thyroid neoplasms, such as clear cell changes, parathyroid neoplasms and clear cell metastases from different sides of the body, especially in RCC [1]. In our present case report, we considered RCC with clear cell and parathyroid neoplasms as well as clear cell follicular carcinoma of the thyroid in the histopathological differential diagnosis. In our patient, the possible signs of follicular carcinoma were outside the capsule in two different areas, pushed the capsule in most of the tumoral area and had a different cytological and histological configuration between inside-outside of the capsule. The absence of glandular formations containing red blood cells in the middle of the vascular structures helped us to differentiate the carcinoma histopathologically. Additionally, thyroglobulin staining during immunohistochemical examination and RCC antibody non-staining with vimentin enabled us to differentiate from RCC. The absence of the space-occupying lesion on radiological examination of the kidney also supported our diagnosis. Non-staining with synaptophysin and chromogranin helped us in the differential diagnosis of medullary carcinoma of the thyroid and parathyroid neoplasms. The clinical, radiological and pathological examinations together allowed us to make the correct diagnosis [4].

## Conclusion

A morphological examination must done first when patients present with thyroid lesions with clear cells. Next, other differential diagnoses including thyroid and parathyroid tissues with clear cytoplasmic changes and clear cell tumor metastases such as RCC must be carefully considered. Our present case report is of interest due to the pathology of a clear cell variant of follicular thyroid carcinoma together with multi-nodular goiter, which is rarely seen.

## Consent

Written informed consent was obtained from the patient for publication of this case report and any accompanying images. A copy of the written consent is available for review by the Editor-in-Chief of this journal.

## Abbreviations

RCC: Renal cell carcinoma; TSH: Thyroid-stimulating hormone.

## Competing interests

The authors declare that they have no competing interests.

## Authors’ contributions

IS made the pathological diagnosis. KP and AI operated on the patient. IG and LD participated in the design of the case report. All authors read and approved the final manuscript.
